# Visual and tactile motion cues enhance the categorisation of novel object shapes

**DOI:** 10.1007/s00221-026-07238-5

**Published:** 2026-02-21

**Authors:** Martina A. Seveso, Rebecca J. Hirst, Alan O’Dowd, Ivan Camponogara, Fiona N. Newell

**Affiliations:** 1 School of Psychology and Institute of Neuroscience, Trinity College Dublin, Dublin, Ireland; 2 Department of Psychology, College of Natural and Health Sciences, Zayed University, Abu Dhabi, UAE

**Keywords:** Object categories, Multisensory perception, Tactile perception, Object motion, Online testing

## Abstract

Object categorisation is a fundamental cognitive process, involving the integration of information across the senses. We investigated, using smartphones, whether visual and tactile motion cues could enhance object category learning and generalisation to novel object shapes. Two categories of similar shapes were associated with specific correlated visual and tactile vibration motion cues. After learning object categories, participants were assessed on categorisation of learned and novel objects across four cue conditions: shape-only, shape-visual motion, shape-tactile motion, and shape-visual and tactile motion. We also assessed if accuracy was influenced by blocked versus interleaved cue-conditions at test. In Experiment 1, we found more accurate categorisation and generalisation when all cues were available at test. In Experiment 2 we replicated this effect even when the reliability of the shape-only cue for predicting category membership was reduced. In Experiment 3, we found that the absence of motion cues during learning removed the benefit of motion cues at test. Overall, our findings suggest that multisensory motion cues benefit the formation of novel object categories and allow for better generalisation. The results have implications for our understanding of the underlying dynamic and multisensory nature of object categories and the predictive role of multisensory features on category formation.

**Supplementary Information:**

The online version contains supplementary material available at 10.1007/s00221-026-07238-5.

## Introduction

Imagine enjoying a peaceful wander through a forest when suddenly, to your horror, something lands on your back. After a while, you recognise that it is an insect and not a leaf falling from a tree. When trying to categorise the “something” as an insect and not a leaf, you might rely on multiple cues, such as the feel of the flutter of the insect’s wings or the scuttling of its legs, while it moves into view. Cues from vision and touch provide partial information, and the perceptual system must decide how these cues should be combined or weighted to support object recognition and category formation. Therefore, understanding how new object categories are learned from multisensory information requires clarifying which features selected, how they are represented, and how they contribute to generalisation beyond learned exemplars.

Previous studies have shown that object category learning is a complex process involving the integration of multiple features to differentiate between object categories as well as generalise from individual exemplars (Goldstone & Hendrickson 2010; Pérez-Gay et al., 2017). These object features may be amodal (e.g., geometric structure or shape) or modality-specific (e.g., tactile vibrations or visual motion) and each type might contribute differently to category formation and generalisation due to distinct representational formats (Proulx et al. 2014). For instance, visual motion patterns and tactile vibrations can each convey unique object features and thus serve as informative cues for category learning through distinct sensory codes. On the other hand, these visual and tactile features may be redundant, such as the feel and sight of an insect’s scuttling legs and integrated to offer an amodal cue to object categorisation (e.g. James & Blake 2004).

Although much is known about unisensory visual (Rosch, 1978) auditory (Griffiths & Warren 2004; Feng et al 2021; Brunel et al. 2013) and tactile (Newell 2004) features on object categorisation, less is known regarding how multisensory cues are used together for category learning and generalisation (Newell et al. 2023). Previous studies have shown that both vision (Peissig & Tarr 2007; Grill-Spector 2003) and touch (Gibson 1962; Lederman & Klatzky 1987; 1990) are sufficient to enable the learning and subsequent recognition of individual objects (Newell et al. 2001; Lacey et al. 2009). Moreover, object information can be efficiently transferred between vision and touch (Yildrim & Jacobs, 2013). For familiar objects, the resulting shared representations across modalities may lead to the formation of more robust object categories than unisensory information alone (Yildirim & Jacobs 2013; Gaissert & Wallraven 2012; Haag 2011; O’Callaghan et al. 2018; Broadbent et al. 2020), highlighting a potential role for multisensory integration in the representation of object categories in memory (Naci et al. 2012).

Despite our knowledge that multiple sensory features contribute to categorisation, it is unclear how these inputs affect the formation of novel object categories. Moreover, our understanding of whether such multisensory categories (i.e., category representations defined by crossmodal as opposed to within-modality features) allow for generalisation to novel exemplars. Indeed, previous studies investigating generalisation from learned multisensory representations of objects have produced mixed results, with some reporting evidence for a benefit of multisensory learning on generalization (Wu et al. 2021), while others report no specific benefit of multisensory information on categorisation performance (Edmunds et al. 2020; Roark et al. 2021, 2023; Sun et al. 2023; Atkin et al. 2023; Li & Deng 2023; Roark 2024; O’Dowd et al., 2025). Some of the discrepancies across these studies may be due to the amount of prior knowledge available about the objects themselves, the extent to which sensory information is correlated across modalities during learning, or the relative distinctiveness or predictability of each sensory cue to category membership.

Objects in the real world are not only defined by their shape, but also their motion (e.g. Newell, Wallraven & Huber., 2004; Robert et al. 2023). When information from two sensory modalities shares temporal properties, such as synchronous movement, they are more likely to be combined (Parise & Ernst 2016), which may serve to enhance any benefit of multisensory information for category learning. Within such a multisensory process, information gathered about the movement of an object through vision (Robert et al. 2023; Shatek et al. 2022) or touch (Gaissert & Wallraven 2012; Simões-Franklin et al. 2011; Sumser et al. 2024) can play a fundamental role in category formation. Visual object movement can facilitate the recognition of novel objects (Stone 1999; Newell et al. 2004; Setti & Newell 2010) compared to static conditions and the neural substrates underpinning tactile and visual object motion appear to be shared (Chan et al 2010; Amemiya et al. 2017). Within the tactile modality, dynamic motion cues are often received in the form of vibrations, which can provide valuable information about specific object properties. In this regard, studies on vibrotactile discrimination suggest that touch can enhance discrimination (Mahns et al. 2006; Verrillo et al. 1969), particularly when visual temporal cues alone are unreliable or ambiguous (e.g., Pomper et al. 2014; Hirst et al., 2025). Tactile stimulation is quickly detected on the skin, and passive exposure to moving tactile information, such as vibrations or flutterings, can help discriminate objects (Fleming et al. 2013; Fleming 2017; Ryan et al. 2021; Shao et al. 2016; Ziat 2023). Because visual and tactile motion cues contribute to object recognition and discrimination, we can assume they also contribute to object categorisation, but this has not hitherto been investigated. Building on this, the present work investigates whether motion-related cues can facilitate not only category learning but also generalisation across novel exemplars.

In the following three experiments, participants learned to categorise novel shapes presented with visual and tactile motion cues (Experiments 1 and 2), or with only object shapes presented during learning (Experiment 3). We manipulated the cue informativeness of category membership and examined how the number and type of cues available affected both learning and generalisation to novel objects. Furthermore, we compared blocked versus interleaved trial presentation at test, supported by previous evidence showing that while blocked trials reinforce within-category featural similarities, interleaving promotes discriminative contrast across categories (Carvalho & Goldstone 2015; Kost et al. 2015) and may enhance long-term retention and cross-modal category induction (Ge et al. 2021; Abel 2023). However, interleaving can increase cognitive load, particularly during cue-switching across modalities (Carvalho & Goldstone, 2014), and uncertainty in cue prioritisation (Carvalho & Goldstone 2019). Thus, in a multisensory context, interleaving trials may either strengthen or compromise categorisation and our manipulation of trial presentation type allowed us to examine these effects.

### Public significance statement

This study shows that combining visual and tactile (vibration) motion cues supports both learning and generalisation of object categories more efficiently than relying on shape alone. Additionally, the way we learn influences our ability to generalise categories to novel objects. These findings highlight the multisensory nature of how we form object categories and suggest applications for designing multisensory educational tools, haptic interfaces, and virtual environments.

### Experiment 1

Our first experiment was designed to explore whether the concurrent availability of visual and tactile motion cues enhances object category formation and categorisation performance. Building on prior research on the perception of moving objects (e.g., Setti & Newell 2004; Chan et al. 2010), we hypothesised that the combination of visual and tactile motion cues with object shape would improve object categorisation and generalisation to novel exemplars relative to static shape alone. We therefore predicted higher categorisation accuracy, including for novel exemplars, in conditions in which all cues were available relative to conditions in which one or more sensory cues were missing. To allow us to explore our secondary factor (blocked versus interleaved trials), we tested this prediction using trials that were either blocked by cue condition (Experiment 1A) or interleaved (Experiment 1B).

## Method

### Participants

We report how we determined our sample size, all data exclusions, all manipulations, and all measures in the study. All participants were recruited using Prolific (https://www.prolific.co/), based on the following inclusion criteria: fluency in English, normal or corrected-to-normal vision and no hearing impairments. An a priori power analysis for each of the groups was performed in PANGEA (Westfall 2016, v0.2) for a within-subjects design. The advised minimum sample size for achieving 80% power to detect the effect of interest (see Analysis below) with a Cohen’s *d* of 0.3 was 32 participants per experimental design (blocked or interleaved). We initially recruited 155 participants of whom 110 (71%) successfully reached the learning criteria. Of these participants who learned the categories, a further 33 failed to perform above chance in the test (29% of learners) and their data were not included in any subsequent analyses (see Fig. S1 in Supplementary Materials for further details). While this attrition rate may appear high, it is in line with findings from other studies on successful categorisation performance in laboratory settings (e.g. Smith et al. 2014; Roark & Chandrasekaran 2023). In total, 77 participants (mean age = 38.83 years, SD = 10.97; 52% female) completed the experiment online with 45 participants allocated to the blocked design (Experiment 1A) and 32 to the interleaved design (Experiment 1B). For all experiments, all participants were naïve to the purpose of the study and were compensated at a rate of £9.00 per hour. The study was approved by the Trinity College Dublin, School of Psychology Research Ethics Committee (approval number-SPREC102020-50) and complied with GDPR data protection legislation.

### Stimuli

**Fig. 1:**
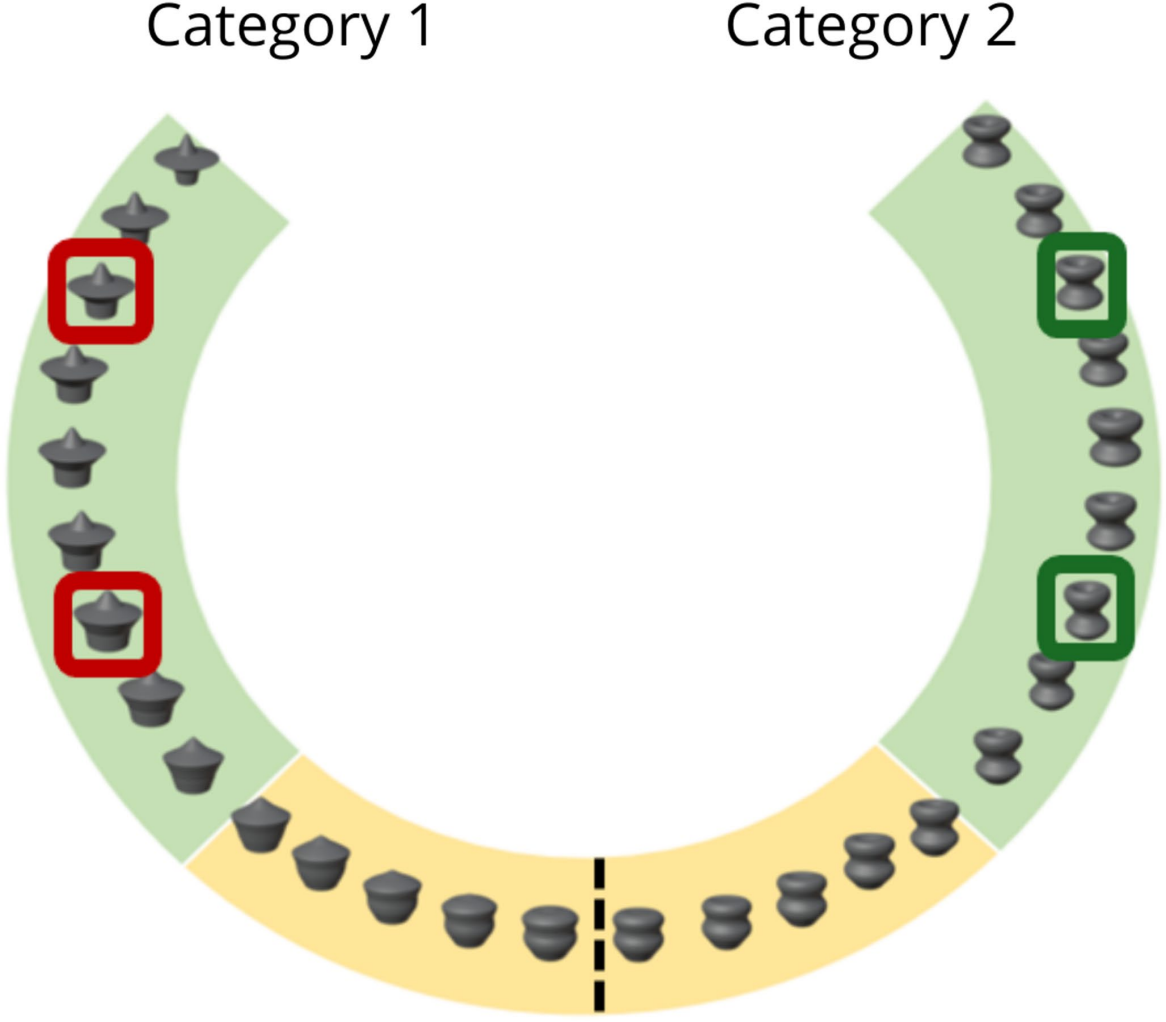
An illustration of the ‘shape space’ of object stimuli used in the Experiments.* Note:* The shape space was adapted from a previous circular shape space described by Li et al., (2020). The objects are arranged within the shape space such that neighbouring objects are equal in angular distance from each other. The exception to this is the object stimuli at the two endpoints of the shape space. Participants were trained to categorise objects highlighted in green (Category 1 and Category 2; 9 shapes per category) and tested using these learned objects as well as a set of novel object stimuli highlighted in yellow (5 per category). The dashed line depicts the (arbitrary) category boundary of this shape space used in the Experiments. The green and red framed stimuli are relevant to the design of Experiment 2 only. See Methods for further details

We created 28 3D novel shapes derived from a shape space as shown in Fig. 1. The design of our shapes was based on a morphing process described elsewhere (Li et al. 2020), and the design of the shape space was adapted from a circular shape space described in previous studies (Li et al. 2020; O’Dowd et al., 2025). As such, all object shapes were arranged in a circular space with equal angular spacing between neighbouring shapes, except for object shapes at each end of the shape space. These object shapes were used as stimuli throughout all three experiments.

We used 3D modelling software (Blender Foundation, 2023, v3.5.0, www.blender.org) to render each 3D object stimulus used in the present experiments. The objects were created using the pipeline described in detail under Supplementary Materials (Section S1). During a trial, the visual presentation of each object was followed by a visual mask. These individual masks were created by scrambling the image of each object shape using a MATLAB script (MathWorks 2023). All object stimuli and scripts for generating the visual masks are available on the Open Science Framework page for Experiment 1, 10.17605/OSF.IO/S369C or https://osf.io/s369c/?view_only=9015e8935be24628b5b15596b8eb6271.

We allocated an arbitrary category boundary to the middle of the stimulus shape space (see Fig. 1), such that 14 objects were allocated per category. Within each category, shapes were highly similar, and shape similarity was the main cue for categorisation. Of the 14 objects per category, 9 objects were selected at the extreme point of the category for the learning session (green highlighted object shapes in Fig. 1) and 5 objects per category, closer to the category boundary, were used for generalization testing (yellow highlighted object shapes in Fig. 1). The use of object stimuli nearest to the category boundary for generalisation was to avoid using clearly distinguishable shapes, which would have reduced the difficulty of the task and possibly obscured any generalization effects.

**Fig. 2:**
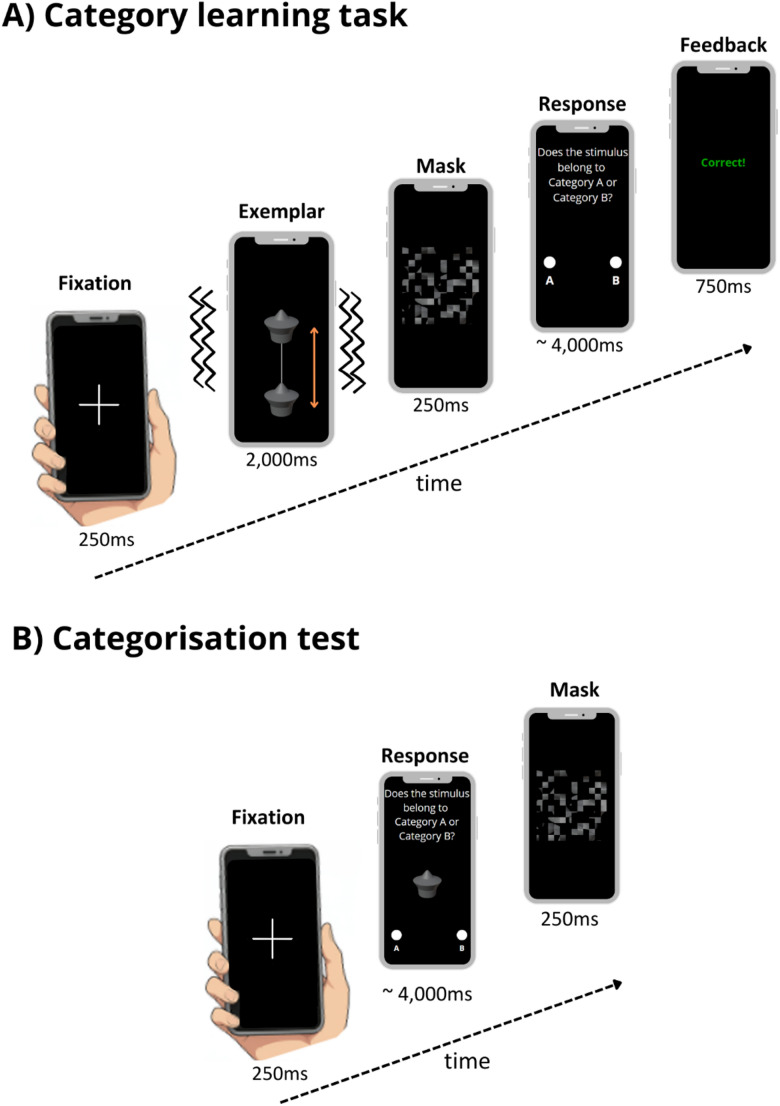
A schematic illustration of the sequence of events from stimulus presentation to the response display used in **A** the learning session and **B** the categorisation test in Experiments 1 and 2. *Note:* Only android mobile phones were used to in the experiment. The timeline of events starts from the left image and proceeds towards the right image for each of the learning and test examples. Wavy lines represent tactile vibrations which were correlated with the visual motion patterns. The categorisation test **B** depicts a trial from the Shape-only (S_v_) condition. See text for details on the trial structure

Each rendered 3D-object was animated based on one of four different visual motion patterns; swing, jump, roll or shake, using the Workbench Engine. The motion patterns were chosen based on type (e.g., smooth or abrupt movement) and reference (e.g., movement of the object’s horizontal or vertical axis). All movement sequences had a duration of 2 s. For the animation, each object stimulus was presented against a background consisting of two grey walls and a grey floor. The light source illuminated the scene from above, creating an object shadow on the floor to aid depth and motion perception. The individual moving objects were then extracted and used as stimuli in the experiment to be displayed against a black background on a mobile phone screen. The entire screen was used to display the objects and response options (see Fig. 2 for an illustration).

Tactile stimuli were delivered via the navigator.vibrate() function; a method of delivering tactile stimuli remotely via android smartphone browsers. This method is described, tested and validated elsewhere (Hirst et al., 2025), in short it allows the presentation of a vibration pulse sequence by specifying binary on/off commands, it does not allow for manipulation of vibration amplitude. Our tactile stimuli therefore consisted of a vibration pulse sequence derived from the visual motion pattern of each stimulus. This was achieved by extracting the temporal dynamics of visual motion for each movement (i.e., frequency and amplitude of movement) and mapping this onto an audio waveform with corresponding frequency and amplitude. The audio waveform was analysed using a 100 ms rolling average to smooth fluctuations in amplitude, and a threshold was applied at 40% of the maximum amplitude to detect markers used to define pulse onset (“on” or vibration) and offset (“off” or pause) events. The resulting vibration patterns therefore closely mirrored the structure and timing of the visual motion stimuli with high temporal precision. A custom Python code (see OSF project page) ensured that the output format retained the timing and structure of the original input and was compatible with the PsychoPy tactile delivery system. The validity of our tactile motion stimuli was supported by the results of pilot tests requiring participants to match vibration pulse sequences with their visual counterparts as well as an assessment of synchrony judgements based on tactile-only and bimodal (i.e., visual motion paired with tactile vibration) stimuli (see Supplementary Materials, Table S1 for results).

### Design

We used three different cues for categorisation in our experiments: visual object Shape (S_v_), visual Motion (M_v_) and tactile Motion (M_t_), summarised in Table 1. The experiment was structured around two main sessions: a learning block (with feedback) followed by a test block (without feedback). During the learning session, all object shapes were displayed with visual and (correlated) tactile motion therefore, category membership was defined by all three cues equally (i.e. S_v_M_vt_). Each shape was associated with one of 4 visual motion patterns (swing, jump, roll, or shake), which were randomly assigned to the shapes across participants (but the shape-motion associations remained constant for each participant). The group of shapes assigned to each category were counterbalanced across participants.

**Table 1:** Summary of the different cues used for object categorisation in all experiments

Object features	Modality	Category cue *(abbreviation)*
Shape (S)	vision (v)	S_v_
Motion (M)	vision (v)	M_v_
Motion (M)	tactile (t)	M_t_
Motion (M)	vision and tactile (vt)	M_vt_

At test, we conducted two versions of the experiment in which trials were either blocked (i.e., Experiment 1A) or interleaved (i.e., Experiment 1B), with different participants taking part in each experiment. Both versions were based on the same within-subjects, fully factorial design with cue condition (4) and exemplar type (2) as factors. The four levels to the cue condition were: shape only (S_v_); shape with visual motion (S_v_M_v_); shape with tactile motion (S_v_M_t_) and shape with both visual and tactile motion (S_v_M_vt_). The exemplar factor had two levels: learned or novel. Our primary outcome measure of interest in the test phase was categorisation accuracy.

### Procedure

The experiment was built using PsychoPy (Pierce et al., 2019, 2022, v2024.2.3) and delivered online through Pavlovia (https://pavlovia.org/) on Android mobile devices. To mask the sound of the vibration stimuli, continuous Brown noise was presented throughout the study. Brown noise was selected due to its low frequency weighted spectrum and because it has been commonly applied as a neutral sound-mask in experimental and environmental studies (Hirst et al., 2025; Hongisto et al. 2017). The level of the Brown noise was individually calibrated via a method-of-adjustment procedure: participants listened to a repetitive 200-ms vibration stimulus (500 ms. ISI) together with continuous Brown noise and adjusted the volume on their phone until the vibration sound was no longer audible. This volume level remained constant for the entire experiment ensuring that any potential influence of the noise mask could not account for condition-specific effects. Following this method of adjustment procedure participants began the learning phase (as shown in Fig. 2A). Each trial in the learning started with a 250 ms fixation cross, followed by the object stimulus presentation for 2 s. Here, the three cues were combined as a visually moving object presented in synchrony with tactile vibrations. The object stimulus was followed by a 250 ms visual mask. Responses were recorded only once the object stimulus had been presented, i.e. after 2 s had elapsed, corresponding to the duration of a full motion pattern. To respond, the participant was presented with a screen indicating the response options (i.e., Category A” or “Category B”). A response triggered the offset of the response screen, but if a response was not made within 4 s the task progressed automatically, and no response was recorded. Feedback on both accuracy and response time was presented for 750 ms at the end of each learning trial, e.g., green “Correct” or red “Incorrect” and “Too slow! Please respond faster” respectively). An accuracy threshold of 75% was required at the end of the learning session to continue to the categorisation test. If participants failed to reach this threshold within 3 repetitions of the trials, the study was terminated. There was a maximum of 54 trials (18 object shapes, with no more than 3 repetitions) during learning. Trial order was fully randomised across participants.

In the test phase, 28 stimuli (14 per category) were presented, including the 9 previously learned and 5 novel exemplars per category. Each test trial began with the presentation of a 250 ms fixation cross, followed by an image of a stimulus (depending on cue condition) alongside response options, participants were given 4 s maximum to make a response before the task progressed automatically (see Fig. 2B). A visual mask appeared for 250 ms after the cue to indicate the end of each trial. No feedback was provided at test. Each stimulus was presented under one of the four cue conditions (S_v_, S_v_M_v_, S_v_M_t_, S_v_M_vt_) and participants were instructed to categorise each stimulus as either ‘A’ or ‘B’ as accurately and quickly as possible. In Experiment 1A, cue condition was blocked at test, such that one block included trials in which stimuli were displayed from one cue condition only (e.g., S_v_ only). The blocks were presented in a random order across participants. In Experiment 1B all trials were presented as interleaved and in a random order across participants. For both Experiment 1A and 1B, no feedback was provided during the categorisation test. Each participant took approximately 18 min to complete the experiment.

### Analysis

The data were analysed with R via RStudio (R Core Team 2021). To assess the effect of cue condition and exemplar learning on categorisation performance, we fitted a generalised linear mixed-effects model (GLMM) on the raw participants’ responses (0, 1) with a binomial distribution and logit link function. Themodel included cue condition (S_v_, S_v_M_v_, S_v_M_t,_ S_v_M_vt_), category exemplar (learned or novel), and their interaction as the predictors of interest. To establish statistical significance, likelihood ratio tests (type-II) were performed to compare the fit of the model with and without that predictor of interest, the model fit was evaluated via AIC, BIC, and log-likelihood values. ‘Participant’ was included as a random intercept, and a random slope for ‘exemplar’ was also included to account for within-subject variability in the effect of exemplar on categorisation performance.^1^ Models were fitted using the ‘lme4’ package (Bates et al., 2015). Post-hoc comparisons were conducted using the ‘emmeans’ package (Lenth Russell et al. 2022) and the Bonferroni correction was applied to correct for multiple comparisons (in which case the corrected *p-*values are reported). Fixed effects from the final model were converted from log-odds to predicted probabilities using the inverse logit transformation to facilitate interpretation (Muller & MacLehose 2014).

## Results

**Fig. 3:**
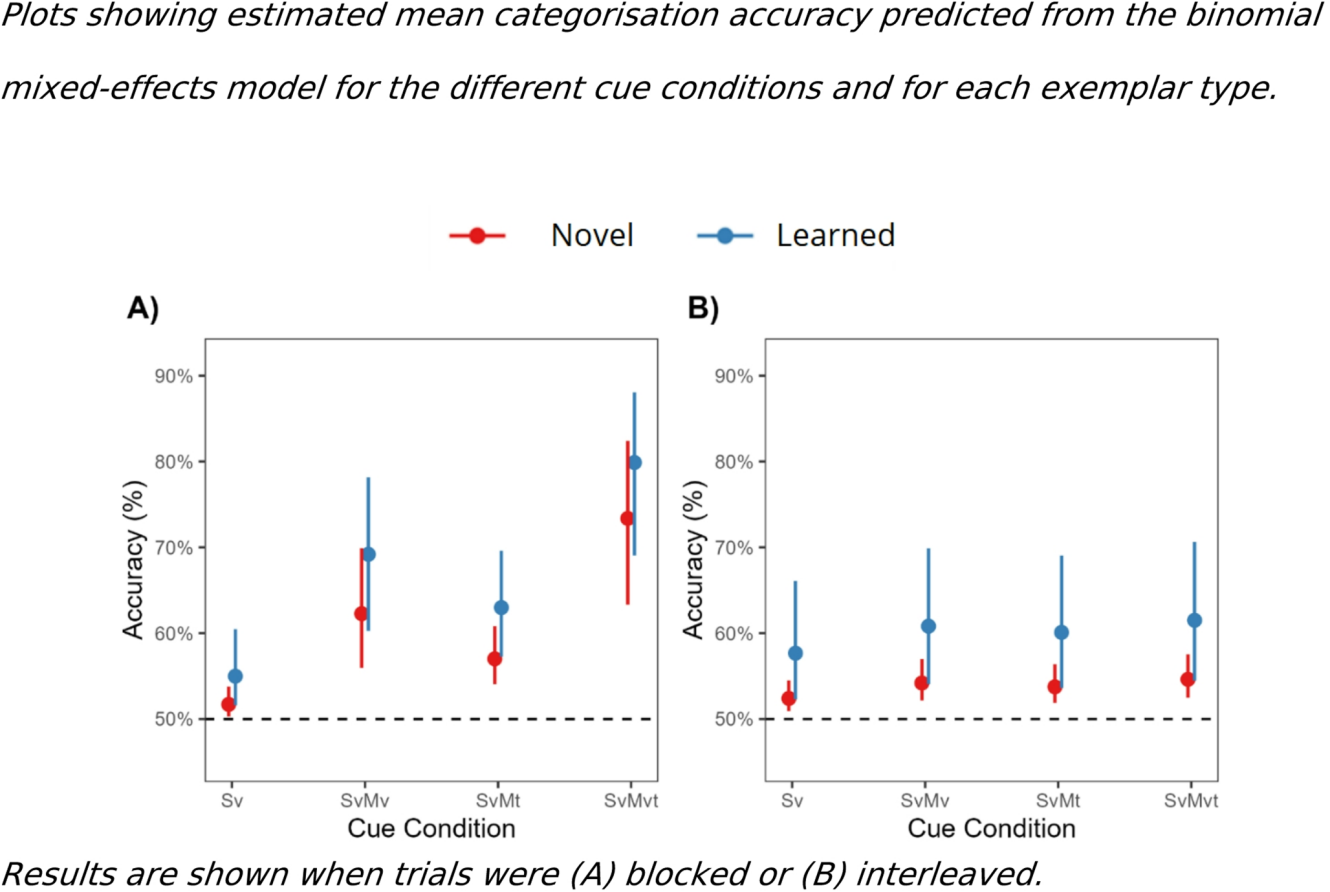
Plots showing estimated mean categorisation accuracy predicted from the binomial mixed-effects model for the different cue conditions and for each exemplar type. Results are shown when trials were **A** blocked or **B** interleaved. *Note:* Error bars represent 95% confidence intervals. Dashed line indicates chance level (50%)

The mean categorisation performance across each of the cue conditions and exemplar types is presented in Fig. 3 for the blocked (A) and interleaved (B) versions, respectively. In Experiment 1A, in which trials were blocked at test, likelihood ratio tests indicated statistically significant main effects of cue condition (*χ*2(3) = 26.50, *p* < 0.001) and exemplar type (*χ*2(1) = 11.80, *p* < 0.001) on categorisation accuracy. However, the cue condition by exemplar type interaction did not significantly contribute to the model (*χ*2(3) = 6.40*, p* = 0.094). Post-hoc comparisons for the main effect of cue condition confirmed that categorisation accuracy was significantly higher for the S_v_M_v_ odds ratio, OR = 0.395, 95%CI [0.224,0.698], *p* < 0.001), S_v_M_t_ (OR = 0.565, 95%CI [0.376, 0.848], *p* = 0.001), and S_v_M_vt_ (OR = 0.209, 95%CI [0.101,0.430],* p* < 0.001) compared to the S_v_ condition. Moreover, categorisation accuracy was significantly lower in both the S_v_M_v_ (OR = 0.528, 95%CI [0.330, 0.845], *p* = 0.002) and S_v_M_t_ (OR = 0.370, 95%CI [0.208,0.657],* p* < 0.001) conditions, compared to the S_v_M_vt_ condition (see Fig. 3A). No significant differences were observed between the S_v_M_v_ and S_v_M_t_ conditions (OR = 1.429, 95%CI [0.871, 2.344], *p* = 0.343). Post-hoc comparisons on the main effect of exemplar type suggested that participants’ categorisation performance was less accurate for the novel (75%) compared to the learned (82%) object exemplars (OR = 0.67, 95%CI [0.538,0.834]*, p* < 0.001; see Fig. 3A).

Likelihood ratio tests on the performance in the interleaved version of the Experiment 1B suggested similar effects. We found statistically significant main effects of cue condition (*χ*2(3) = 12.20, *p* = 0.007) and exemplar type (*χ*2(1) = 8.08, *p* = 0.005) in the model predicting categorisation accuracy. Again, the cue condition by exemplar type interaction did not significantly contribute to this model (*χ*2 (3) = 1.47, *p* = 0.69). Post-hoc comparisons for the cue condition effect revealed that accuracy was significantly lower in the S_v_ condition compared to the S_v_M_v_ (OR = 0.806, 95%CI [0.654,0.993], *p* = 0.039) and S_v_M_vt_ conditions (OR = 0.772, 95%CI [0.627,0.950], *p* = 0.006), as shown in Fig. 3B. However, we failed to find a significant difference between the S_v_ and the S_v_M_t_ condition (OR = 0.845, 95%CI [0.686, 1.040], *p* = 0.194). Additionally, no significant differences were found between the two-cue conditions (S_v_M_v_ vs S_v_M_t_: OR = 1.05, *p* = 1); and there was no benefit found to the S_v_M_vt_ condition relative to either the S_v_M_v_ (OR = 0.958, *p* = 1) or S_v_M_t_ conditions (OR = 0.914, *p* = 1). Regarding the exemplar type, categorisation accuracy was significantly lower for novel (65%) compared to learned (76%) exemplars (OR = 0.597, 95%CI [0.422,0.844], *p* = 0.004), see Fig. 3B. Finally, a one-sample t-test showed that accuracy in the S_v_ condition was significantly above chance for both Experiment 1A (M = 0.61, SD = 0.23, t (44) = 3.21, *p* = 0.002), and 1B (M = 0.63, SD = 0.18, t(30) = 4.09, *p* < 0.001).

## Discussion

Experiments 1A and 1B indicated a benefit of having all learned cues, S_v_, M_v_, M_t_, available at test, relative to when only the shape of the object exemplars was available. Categorisation performance was least accurate, although above chance, in the shape-only cue condition, suggesting that shape similarity alone did not result in robust categorisation. Performance to novel exemplars was further affected, although our data suggested that generalisation occurred even in the shape-only cue condition. Interestingly, while categorisation performance was affected by the number of available cues, the data suggest no specific benefit for any one sensory modality, at least when trials were blocked. In other words, performance across both two-cue condition (S_v_M_v_ and S_v_M_t_) was equally accurate, suggesting that visual and tactile motion contributed to category formation in a similar way. However, this result was influenced by the randomization procedure: in the interleaved trials tactile motion alone (S_v_M_t_) did not improve performance relative to the object shape-only cue (S_v_), unlike in blocked conditions where both tactile (S_v_M_t_) and visual (S_v_M_v_) motion independently improved performance relative to static conditions.

In contrast, we found a stronger advantage for cue combination in the blocked (Experiment 1A) trial presentation. Under blocked presentations, there was a greater distinction in performance across the different cue conditions. This finding suggests that task consistency may support more effective use of the cues, whereas interleaving trials may add uncertainty reducing the observed benefit of information from combined cues on categorisation.

As expected, accuracy was consistently better for learned relative to novel exemplars, and there was no influence of trial presentation on the categorisation benefit for learned exemplars (7% and 11% difference between learned and novel exemplars in the blocked and interleaved versions, respectively), suggesting little effect of task demands. As noted earlier, exemplars that are positioned closer to the category boundary are typically more difficult to categorise than those that are positioned further from the boundary (Newell & Bülthoff 2002). Therefore, the difference in performance between learned and novel exemplars may be influenced by stimulus position within the category shape space as well as exemplar novelty. Future research could help tease apart the relative contributions of exemplar position in shape space and frequency of exposure on categorisation. Interestingly, the effect of cues did not differ between learned and novel exemplars in either version of the experiment, suggesting that the benefit afforded by multisensory motion cues is no higher for novel compared to learned exemplars.

The number of participant data sets included in the analyses was lower than the initial number of participants recruited. This was necessary for several reasons. First, we required that the participants first reach a learning rate of 75%, which is a reasonable rate given the number of factors involved in the experiment and is consistent with other studies on categorisation. Second, we also required that participants’ performance was greater than chance level across the task. A number of participants failed to meet this requirement although they had successfully learned to categorise the objects. Although our overall attrition rate from initial recruitment appears high, it is not inconsistent with other studies of rates of successful categorisation performance in the lab (Smith et al. 2014; Roark & Chandrasekaran 2023) or indeed in reports of dropouts from online testing in behavioural studies (e.g. Peer et al. 2022; although notably, data collected via Prolific is often considered of high quality).

Together, these findings suggest that a combination of cues from different sensory modalities can facilitate object categorisation and generalisation relative to object shape alone. Given that the visual and tactile motion cues to category membership were few in number and consistently reliable, our result that tactile motion did not benefit categorisation relative to shape-only cues in the interleaved trials might be considered surprising. Because of their consistency, both the visual motion and tactile motion cues might be expected to dominate categorisation performance relative to the shape cue alone. Since categorisation performance was better than chance to the shape cue (S_v_) alone we can infer that shape was informative for categorisation. Indeed, the presence of each motion cue during test appears to have influenced categorisation performance in an additive manner, at least when trials were blocked. Given this, in Experiment 2, we aimed to further explore the contribution of the shape cue by manipulating its informativeness relative to other cues on categorisation.

### Experiment 2

Perceptual similarity may support the formation of categories, such that similar shapes are grouped together under a single category label (e.g., Nosofsky 1986; Goldstone 1994), even if encoded through touch (Cooke et al. 2007). However, in the real world, information about an object’s shape can be affected by environmental factors such as distance, occlusion, lighting or viewpoint (e.g., Newell 1998), thus reducing the reliability of shape similarity for determining categorisation. Consequently, other cues to categorisation may become salient such as the way an object moves (Newell et al. 2004; Blake & Shiffrar 2007). To investigate this, we reduced the informativeness of the object-shape similarity as a cue on category membership. Importantly, we maintained both the visual and tactile motion cues as fully predictive of category membership, as in Experiment 1. Consequently, we hypothesised that participants’ categorisation performance, to both learned and novel exemplars, should be mainly influenced by motion cues. As in Experiment 1, we are testing this in both blocked (Experiment 2A) and interleaved (Experiment 2B) test conditions.

## Method

### Participants

An a priori power analysis was performed using PANGEA v0.2 (for a within-subjects design), which indicated that a minimum of 32 participants for each of the blocked and interleaved versions of the experiment was required to achieve 80% power to detect an effect size of 0.3 (Cohen’s d) for the effect of interest. All participants were recruited using Prolific (https://www.prolific.co/), and inclusion criteria were fluency in English, normal or corrected-to-normal vision and no hearing impairments. We initially recruited 85 participants, of whom 76 (i.e. 89%) successfully learned the categories. Of the learners, 10 participants failed to score above chance in the categorisation test and their data were not included in subsequent analyses. Following these exclusions, a total of 66 participants (mean age = 39.72 years, SD = 10.1; 48.5% female) completed Experiment 2, with 33 assigned to the blocked version (Experiment 2A) and 33 to the interleaved version (Experiment 2B).

### Stimuli

The same stimulus set, and motion types described in Experiment 1 were used. However, we used a different arrangement of the object shapes across categories to reduce object shape similarity as a cue to category membership. To that end, we reassigned two out of the nine exemplars in each category to the opposite category. For example, referring to Fig. 1, object shapes highlighted in red, which were members of the same object category in Experiment 1 (e.g., Category A), were now assigned to the opposite category (e.g., Category B) during learning (and likewise for object shapes highlighted in green). Thus, only 78% of the neighbouring shapes were assigned to one category in the current experiment. The subset of exemplars that were novel but included in the test was the same as in Experiment 1.

### Design and procedure

Experiment 2 followed the same design and procedure as described in Experiment 1 with the same four cue conditions (S_v_, S_v_M_v_, S_v_M_t_, S_v_M_vt_; see Table 1) and two exemplar levels (learned and novel). As in Experiment 1, trials were either blocked by cue (Experiment 2A) or interleaved (Experiment 2B) and participants were pseudo-randomly assigned to each version of the experiment.

## Results

The main analysis procedure was identical to that of Experiment 1.

**Fig. 4:**
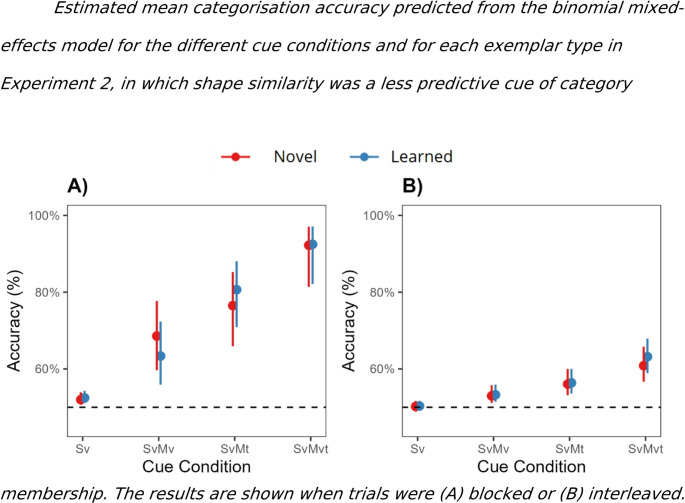
Estimated mean categorisation accuracy predicted from the binomial mixed-effects model for the different cue conditions and for each exemplar type in Experiment 2, in which shape similarity was a less predictive cue of category membership. The results are shown when trials were **A** blocked or **B** interleaved.* Note:* Error bars represent 95% confidence intervals. Dashed line indicates chance level (50%)

Categorisation performance across the four main cue conditions and each learned or novel exemplar condition is presented in Fig. 4. First, categorisation performance on the blocked trials (Experiment 2A) was analysed using a likelihood ratio test, which compared the full model, which included the interaction between cue condition and exemplar type, to a reduced model without the interaction term. The comparison indicated that the full model significantly improved the fit of the data (*χ*2 (3) = 9.09, *p* = 0.028), suggesting that the interaction between cue condition and exemplar type contributed to categorisation accuracy (Fig. 4A). There were significant main effects of both the cue condition and exemplar type on categorisation accuracy. Post-hoc comparisons revealed that categorisation accuracy was significantly lower in the shape-only (S_v_) condition compared to all the combined cue conditions. Specifically, accuracy to the S_v_ condition was significantly lower than to the S_v_M_v_ condition (OR = 0.570, 95%CI [0.306, 1.064], *p* = 0.018), the S_v_M_t_ condition (OR = 1.589, 95%CI [0.987, 2.557], *p* = 0.013), and the S_v_M_vt_ condition (OR = 4.832, 95%CI [1.911, 12.222], *p* < 0.001). As in Experiment 1, no significant differences were found between the S_v_M_v_ and S_v_M_t_ conditions (*p* = 1), nor between each of these two-cue conditions and the three-cue condition (S_v_M_v_-vs-S_v_M_vt_, *p* = 1; S_v_M_v_-vs-S_v_M_vt_, *p* = 1). There was a main effect of exemplar type with better performance to learned (76%) over novel (65%) exemplars (OR = 0.597, 95% CI [0.422, 0.844], *p* = 0.004). The significant interaction between cue condition and exemplar type was driven mainly by performance between each of the two-cue conditions and the S_v_ condition (see Fig. 4A): performance was better to the S_v_M_v_ than S_v_ condition for the learned (OR = 0.570, *p* = 0.018), but not novel (OR = 0.770, *p* = 0.282) exemplars and, similarly, performance was better to the S_v_M_t_ than S_v_ condition for learned (OR = 1.589, *p* = 0.013) but not novel (OR = 1.218, *p* = 0.282) exemplars. Performance was better to the S_v_M_vt_ condition than the S_v_ condition for the novel version only (OR = 4.658, *p* < 0.001).

We then conducted likelihood ratio tests on performance when trials were interleaved (Experiment 2B) which indicated a main effect of cue condition (*χ*2 (3) = 276.72, *p* < 0.001) but not of the exemplar type (*χ*2(1) = 0.953, *p* = 0.329) in the model predicting categorisation accuracy, as shown in Fig. 4B. Furthermore, the cue condition by exemplar type interaction did not significantly contribute to this model (*χ*2 (3) = 0.73, *p* = 0.866). Post-hoc comparisons indicated that categorisation accuracy was significantly lower in the S_v_ condition compared to the S_v_M_v_ condition (OR = 0.617, *p* < 0.001), the S_v_M_t_ condition (OR = 0.455, *p* < 0.001), as well as the S_v_M_vt_ condition (OR = 0.299, *p* < 0.001). Moreover, performance was better to the S_v_M_vt_ condition compared to the S_v_M_v_ (OR = 0.484, *p* < *0.0*01) and S_v_M_t_ (OR = 0.657, *p* < 0.001) conditions. In contrast to the results from the blocked trials, performance to S_v_M_v_ was lower than to the S_v_M_t_ (OR = 0.736, *p* < 0.001) condition when trials were interleaved.

Finally, a one-sample t-test showed that accuracy in the S_v_ condition was significantly above chance for Experiment 2A (*M* = 0.61*, SD* = 0.17, *t* (32) = 4.27, *p* < 0.001*),* but not for Experiment 2B (*M* = 0.50, *SD* = 0.06, *t*(31) = − 0.29, *p* = 0.77).

## Discussion

In Experiment 2, we reduced the informativeness of shape similarity as a cue to category membership to investigate whether participants would rely more on motion cues. The results suggest that when only the shape cue was available at test, performance was relatively poor. This was especially the case when trials were interleaved, i.e., when cue availability was less predictable from one trial to the next, relative to the version in which trials were blocked.

Categorisation performance was best when all cue combinations were available across experiment versions (blocked or interleaved). That is, the combination of shape with both motion cues led to significantly better accuracy than object-shape alone, for both learned and novel exemplars. However, in the blocked condition, this advantage did not extend to the two-cue combinations, in the blocked condition, this advantage did not extend to the two-cue combinations (i.e. S_v_M_v_ or S_v_M_t_ vs S_v_ independently) for novel exemplars, which did not significantly outperform the shape-only condition. The significant interaction between cue condition and exemplar type suggests that while all cue combinations supported learning of the trained exemplars, successful generalisation to novel exemplars relied more on the availability of both motion cues together This suggests that when the reliability of shape as a cue to category membership is reduced, generalisation required the full cue-combination context at test.

Interestingly, when trials were interleaved, tactile motion provided a greater benefit than visual motion on both categorisation and generalisation performance. Again, this finding suggests a greater weighting of reliable cues, such as motion over shape. The relative benefit of tactile over visual motion may be due to the easier combination of visual motion with, and therefore segregation of tactile motion from, the object shape (see, Chen & Spence 2017).

In Experiment 1 and 2, the shape cue was always presented with motion cues during learning; therefore, it was unclear to what extent shape itself influenced learning and consequent categorisation and generalisation performance at test. Indeed, performance was consistently worse to the shape-only cue condition at test, and we attribute this performance to the absence of the other learned cues. In the following control experiment, we investigated the specific contribution of visual object-shape as a cue to category learning.

### Experiment 3

In Experiment 3, participants learned categories defined solely by object shape (static images) and at test were assessed across the same four cue conditions used previously (i.e., S_v_, S_v_M_v_, S_v_M_t_, S_v_M_vt_). The use of motion cues at test further allowed us to assess the added value of multisensory motion cues beyond shape information on categorisation and generalisation.

## Methods

### Participants

We initially recruited 45 participants online using Prolific, 8 of whom failed to reach learning criteria and a further 7 failed to perform above chance at test and their data were not included in the analyses. Therefore, total of 30 participants (mean age = 37.44 years, SD = 10.10; 48.5% female) successfully completed Experiment 3; the reimbursement rate and inclusion criteria were identical to those of the previous Experiments.

### Stimuli

All the stimuli, and arrangement of shapes into the two categories, were identical to that of Experiment 1(see Fig. 1).

### Design and procedure

The experiment was based on the same design and procedure as described in Experiment 1 with the exception that the category learning task was performed on static object-shape stimuli. Also, we blocked trials by cue condition during the test session (as in Experiment 1A). Both learned and novel exemplars were presented at test in a random order across participants. Overall, the experiment took approximately 16 min for each participant to complete.

## Results and discussion

Likelihood ratio tests indicated statistically significant main effects of exemplar type (*χ*2(1) = 34.95, *p* < 0.001) but not of cue condition (*χ*2(3) = 5.25, *p* = 0.15) to the model predicting categorisation accuracy. The cue condition by exemplar type interaction did not significantly contribute to this model (*χ*^*2*^* (*3) = 2.748, *p* = 0.43). Overall, irrespective of condition, participants categorisation performance was less accurate to the novel (76%) compared to the learned (86%) exemplars (OR = 0.14, 95%CI [0.08,0.24], *p* < 0.001).

**Fig. 5:**
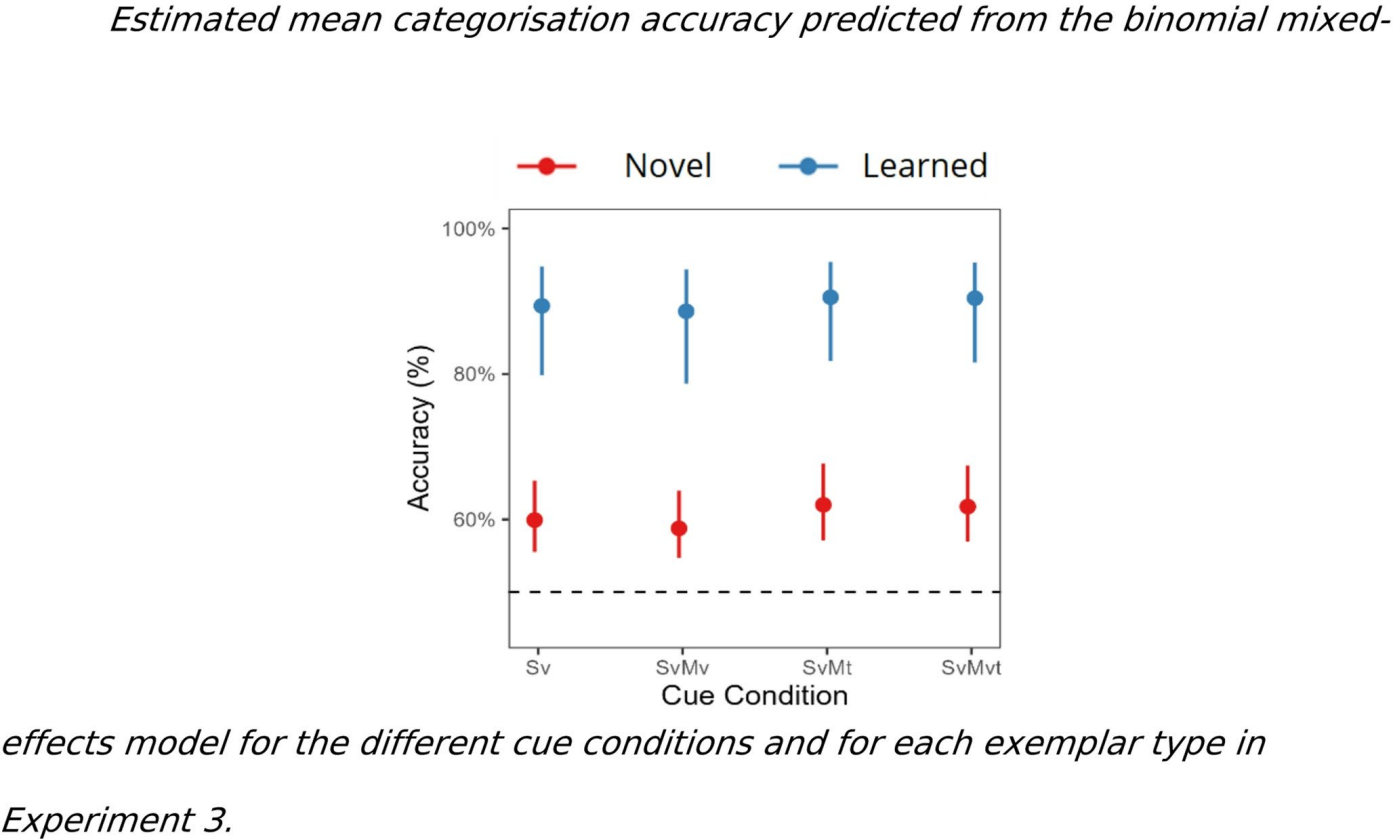
Estimated mean categorisation accuracy predicted from the binomial mixed-effects model for the different cue conditions and for each exemplar type in Experiment 3. *Note:* Error bars represent 95% confidence intervals. Dashed line indicates chance level (50%)

The results suggest that object shape was sufficient for category formation. However, we found that generalisation to novel exemplars was poor, with a drop of approximately 20% accuracy compared to the categorisation of learned exemplars (Fig. 5). Overall, these findings underline the important role of multiple cues during category formation and suggest that the benefit of visual and tactile object motion cues observed in the test session in the previous experiments are contingent on their prior integration into the category structure.

### General discussion

Across three categorisation experiments, delivered remotely via smart phones, we examined whether previously learned multisensory categories, defined by shape, visual motion, and tactile vibration, could support object categorisation and generalisation. The motion cues were correlated across modalities and predictive of category membership (Experiment 1 and 2). Although shape similarity underpinned category membership in Experiment 1, its reliability was reduced in Experiment 2. Overall, the results of both experiments suggested that the addition of visual and tactile motion facilitated both the categorisation and generalisation of exemplars relative to shape-only information. The results of Experiment 3 confirmed that visual shape alone could support category learning in our tasks, although generalisation was poor. In addition, the task format, i.e. whether trials were blocked by cue or interleaved, significantly modulated the observed multisensory benefits: whereas blocked trials (Experiments 1A and 2A) consistently led to higher accuracy and better generalisation, an interleaved presentation (Experiments 1B and 2B) resulted in smaller performance differences between cue conditions and a less robust contribution from tactile cues. Our findings elucidate the featural and context-sensitive nature of multisensory categorisation and demonstrate that the effectiveness of cross sensory cues for categorisation is dependent on cue reliability as well as task structure.

Our results align with previous evidence on the role of cue reliability on perception (Oruç et al. 2003; Helbig et al. 2012; Bankieris et al. 2017) whilst extending this to the realm of category learning. For example, when the inter-object similarity of shapes was reduced as a cue to categorisation (Experiment 2), and motion cues remained fully informative, participants’ categorisation performance was influenced more by the tactile and visual motion cues, particularly under blocked conditions. Our results further support the idea that tactile cues are not merely supplementary but can be integral to the formation of category representations (Yildirim & Jacobs 2013; Gaissert & Wallraven 2012), especially when they are temporally aligned and task-relevant (Nordmark 1978; Heller 1989). Indeed, tactile motion cues played an important role in enhancing categorisation performance in all experiments, suggesting that individuals can strategically adapt their category learning performance to prioritise diagnostic information. Even under interleaved trial presentation (Experiment 2B), performance in combined cue condition remained high, suggesting that redundant motion cues can compensate against uncertainty from the shape cue. However, the pattern of results in Experiment 2B suggests that tactile motion cues may have a stronger impact than visual motion when shape reliability is reduced, especially under interleaved trial presentation. The different effect of motion cues under blocked or interleaved trials suggests that task demands can influence cue combination, including across modalities (Carvalho & Goldstone 2019; Maddox et al. 2006). Although some evidence suggests that interleaved presentation enhances generalisation due to discriminative contrast (Brunmair & Richter 2019; Kornell & Bjork 2008; Carvalho & Goldstone, 2014; 2015), including across modalities (Abel 2023), we found that categorisation performance was weaker for novel than for learned exemplars. Although the reason for this inconsistency is unclear, we suggest that interleaved presentation may either trigger a greater reliance on individual cues or modalities or may impair the retrieval of specific cue-category associations. Under blocked conditions, the motion cues integrated into the object representations may act as contextual supports in memory for categorising the object shape (Duarte et al. 2023; 2025).

In Experiment 3, the substantial (~ 20%) drop in performance to novel exemplars relative to the performance in previous experiments supports the idea that multisensory encoding during learning, rather than test-phase cue availability, is critical for supporting generalisation. This is consistent with evidence that learned multisensory representations subsequently support better generalisation across modalities (Yildirim & Jacobs 2013). Interestingly, there was no benefit for motion cues at test suggesting that unfamiliar, but potentially informative, cues are not automatically used without prior association.

Our ability to categorise objects in the real world is likely to be dependent on a complex interaction between the nature and type of sensory information available, prior knowledge, and other context-dependent factors related to the task. For example, only tactile motion seems to have had a differential influence on categorisation and generalisation performance across task structure (interleaved or blocked trials). Moreover, methodological factors may help explain some of the inconsistencies in the reported findings in the literature (Wang & Zeng 2022; Roark & Holt 2022; Roark et al. 2021; O’Dowd et al. 2025). Consistent with this complexity, our findings suggest that no single mechanism fully explains our data. Future studies are needed to investigate how cue combination benefits learning and subsequent performance. In particularly it would be interesting to know if multisensory cues lead to enhanced learning performance (Shams & Seitz 2008) or if categorisation responses go beyond predictions based on the summation of probability alone (see Nardini et al. 2010). In general, our results support the use of flexible, context-dependent cue combination strategies for learning novel object categories and therefore help contribute to the ongoing debate about the role of multisensory information in object categorisation (Newell et al. 2023).

While our findings show robust benefits on object categorisation and generalisation from the availability of visuo-tactile motion cues, our design does not rule out the possibility that probability summation or crossmodal association played a role. In other words, additional cues may have benefitted categorisation due to extra information and not necessarily solely due to any benefit from feature integration across modalities. Despite this, we believe that our experiment provided optimal conditions for multisensory integration in that the tactile and visual motion cues were always temporally aligned, likely facilitating the effective use of redundant cues across modalities. However, future studies in which, for example, these cues are temporally asynchronous could help determine the extent to which categorisation reflects the integration of temporally coincident cues or simply the presence of additional information.

Our task was designed to enable remote data collection online via mobile devices. Although object perception experiments involving touch typically necessitate in-person testing (see Newell 2004; 2010; Woods & Newell 2004), technological improvements have meant that remote psychophysical testing using different modalities is rapidly increasing (Marin-Campos et al. 2021; Zhao et al. 2022; Hirst et al., 2025; Inuggi et al. 2024). The benefits for remote assessment are well-documented including the recruitment of larger and more heterogeneous participant samples (Grootswagers 2020), and easier longitudinal follow ups. Our work has important practical implications for the remote testing of multisensory category formation. Recent research from our team has validated the use of vibrations for remote testing contexts (Hirst et al., 2025), however to our knowledge no study has yet used this method to deliver complex patterns of vibration stimuli. This is therefore the first study to use complex vibration stimuli, delivered via the browser, to explore multisensory processes. It is of practical note that this method was successful, participants identified the correspondence between vibration patterns and visual stimuli with ease (see Supplementary Material, Table S1) and this enabled us to conduct the current design and share this method for future research. We argue that remote delivery of tactile stimulation through mobile devices is an ecologically valid assessment method given that tactile feedback is increasingly integrated into technology (Muender et al. 2022; Ziat et al. 2022; Zwoliński et al. 2022). Indeed, technological advances now mean that many everyday objects incorporate some form of vibration feedback: you may feel resistance in the steering wheel of your car when changing lanes, the driver’s seat might pulsate to signal danger, or your mobile phone may vibrate to signal an incoming call. Tactile feedback has also been shown to be effective at improving task performance (Fang et al. 2023) and the sense of presence (Huard et al. 2022; Kim et al. 2022). Moreover, delivery through commodity devices has real world implication for gaming or training app design. Despite these potential benefits, few published studies have utilised vibration delivery through mobile phones to study perception, and those that have report similar effects as lab-based studies (Inuggi et al. 2024; Hirst et al., 2025). It is important to note different smartphones may vary in vibration strength and frequency response and that the possibility of these differences having an effect on the results needs to be mitigated. For example, we achieved this by basing the tactile cues on relative timing patterns rather than absolute intensity, and we ensured that the experiment was preceded by a device-specific calibration step. Methodologically, the use of smartphone-delivered tactile cues offers an accessible and ecologically valid way for investigating multisensory interactions, particularly in applied domains such as education, human–computer interaction, and rehabilitation, where redundant sensory input, including from touch, can support cognitive abilities (see Gori et al. 2022).

## Conclusions

The present study investigated whether visual and tactile motion cues enhance the categorisation of novel object shapes, and whether these multisensory categories support generalisation to new exemplars. Across three experiments delivered via mobile phones, we tested whether previously learned multisensory categories—defined by shape, visual motion, and tactile vibration—could support object categorisation and generalisation, across blocked versus interleaved cue-conditions at test. In Experiment 1 all cues were fully predictive of category membership, while in Experiment 2 the reliability of shape information for categorisation was reduced. In Experiment 3, categories were learned with static shape only. When cues were equally reliable (Experiment 1), performance improved when all cues were available at test, and this was replicated when shape reliability was reduced (Experiment 2). Task format modulated these benefits: blocked presentation (Experiments1A and 2A) yielded higher accuracy and stronger generalisation, whereas interleaved presentation (Experiments 1B and 2B) reduced performance differences and weakened contributions from tactile cues. Finally, visual and tactile motion facilitated categorisation and generalisation relative to shape-only learning (Experiment 3). Collectively, these findings demonstrate that multisensory motion cues promote object category formation and generalisation, with effectiveness influenced by cue reliability and task structure. The results have important implications for our understanding of the underlying dynamic and multisensory nature of object categories and the predictive role of multisensory features on category formation. To our knowledge, this is also the first study to use complex vibration stimuli, delivered via the browser, to investigate multisensory processes. These results and methodology provide a valuable resource for future work on tactile and multisensory processing in applied contexts.

## Supplementary Information

Below is the link to the electronic supplementary material.

Supplementary Material 1

## Data Availability

Experiments were preregistered prior to data collection on the Open Science Framework: Experiment-1A, [https://osf.io/2ms48]; Experiment-2A, [https://osf.io/dxwej]; Experiment 3, [https://osf.io/pem72]. All data and both R and Python code supporting the findings of this study are openly available on the Open Science Framework (OSF) at [10.17605/OSF.IO/S369C] for Experiment 1 (A,B), at [10.17605/OSF.IO/VKG7C] for Experiment 2 (A,B), and [10.17605/OSF.IO/CNMHV] for Experiment 3.

## Footnotes

1 *Note:* The structure of the random effect changes in the experiments, due to conversion issues. Experiment 1A has ‘task’ and ‘exemplar’, 1B has ‘exemplar’, 2A has ‘task’ and 2B has none, and Experiment 3 has ‘exemplar’ as random intercept. For more detailed information, refer to the OSF project page.
