# Supplementary material for: Visual and tactile motion cues enhance the categorisation of novel object shapes
Source: Exp Brain Res. 2026 Feb 21;244(4):48. doi: 10.1007/s00221-026-07238-5 (PMC12924856; doi:10.1007/s00221-026-07238-5)
Supplement: Supplementary file 1 — Supplementary Material 1 [file 221_2026_7238_MOESM2_ESM.docx]

#

# Visual and tactile motion cues enhance the categorisation of novel object shapes

Martina A. Seveso^1^, Rebecca J. Hirst^1^, Alan O’Dowd^1^, Ivan Camponogara^2^ and Fiona N. Newell^1^

^1^School of Psychology and Institute of Neuroscience, Trinity College Dublin, Ireland. 
^2^Department of Psychology, College of Natural and Health Sciences, Zayed University, Abu Dhabi, United Arab Emirates.

# Author Note

Correspondence concerning this article should be addressed to Martina A. Seveso, Institute of Neuroscience, Trinity College Dublin, Dublin, Ireland. Email: [sevesom@tcd.ie](mailto:sevesom@tcd.ie), ORCID: <https://orcid.org/0000-0001-6566-4578>.

**Supplementary Information**

***S1 –*** Stimulus design pipeline for *the 3D object models.*

The objects were created using the pipeline (1) each shape was converted from .png to Scalable Vector Graphics (.svg) keeping three different colour levels constant (e.g., white, black, grey); (2) each vector was imported into the 3D-space; (3) the outline was isolated and converted into a mesh; (4)each mesh was rotated along the central vertical axes through the Spin Function in Edit mode (360°, steps-100); (5) each 3D-shape was extracted from Blender. The 3D-space, lighting (point, radius-0.1m,1000W; coordinates:11m,-14 m,6.9m; rotation:40°, 34.8°) and viewpoint (coordinates: 10.9m, -14m, 4m; rotation:70°, 0°) settings were kept constant. Each 3D-object was rendered using the Workbench Engine (28-render samples, Single pass Anti-Aliasing viewpoint; Studio Lighting, Colour Material [dark grey, RGB:107,109,109,254; HEX:#6b6d6d] and Specular Lighting). All the object images were extracted with a resolution of 1080 x 1080 px, scale100%, and presented in a canonical, 3/4view so that the 3D-object and relevant features (e.g., concavities) were visually accessible in the image. The camera angle (coordinates:10.9m, -14m, 4m; rotation:70°, 0°) and lighting (point, radius-0.1m, 1000W; coordinates: 11m, -14 m, 6.9m; rotation:40°, 34.8°) were both held constant.

**Figure S1***– Number of participants who passed each stage of the experiment and final sample per experiment.*


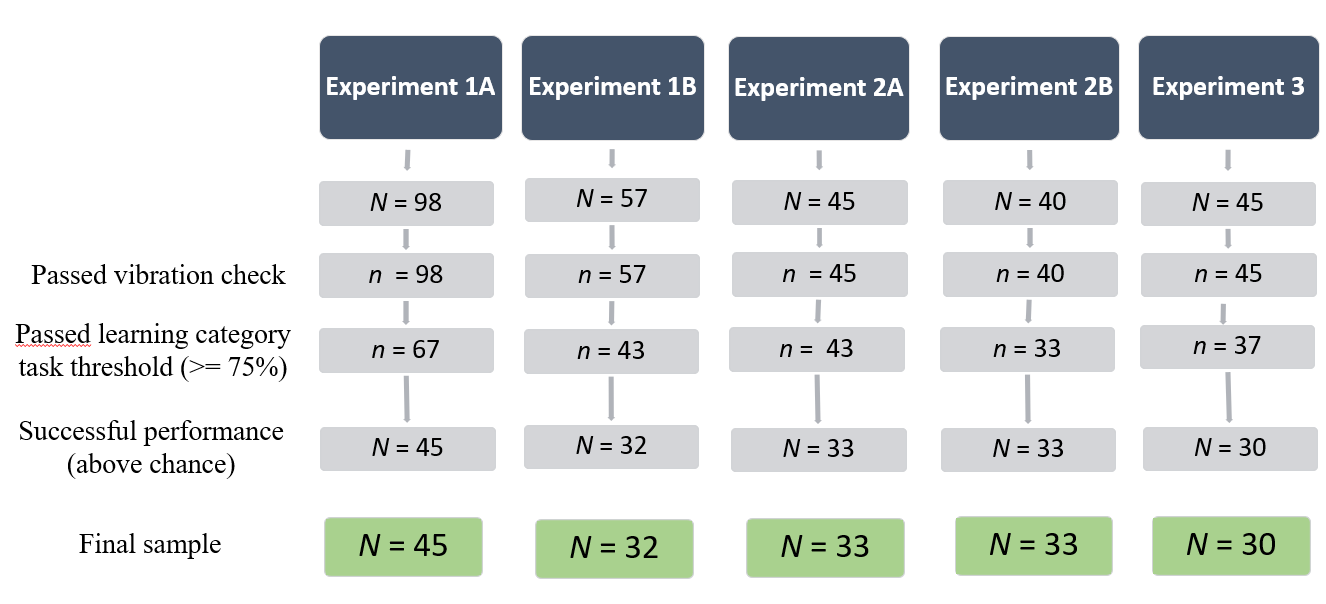


*Note.* The higher exclusion rate in Experiment 1a reflects the fact that the initial 80% learning criterion was too stringent; after observing this, we lowered the threshold to 75% for subsequent participants. All participants recruited (for both thresholds) are included here for transparency.

***Table S1*** *– Results of synchrony assessment pre-test prior to each of the Experiments 1A,1B,2A,2B,3.*

To assess perceived synchrony across three stimulus onset asynchronies (SOAs: 0 ms, 150 ms, 300 ms), we conducted a ratings study (1-5) in which participants rated the perceived synchrony of visual and tactile events (a rating of 5 indicated highly synchronous and 1 highly asynchronous). Separate Welch ANOVAs were conducted for each experiment. Assumption checks revealed violations of normality and homogeneity of variance in all experiments except Experiment 3 (see Table below). Accordingly, Welch ANOVA was used due to its robustness to such violations. The table provides the mean ratings for each SOA per experiment and the analysis of differences across these ratings.

| **Experiment** | **Mean synchronous values across SOAs** | **Shapiro–Wilk (Normality)** | **Levene’s Test (Homogeneity)** | **Welch ANOVA (*F*, df)** | ***p*** | | **Significant Pairwise Differences** |
| --- | --- | --- | --- | --- | --- | --- | --- |
| *Exp. 1A* | 0ms= 3.66  150ms= 3.24  300ms= 3.08 | W = 0.910,  p < .001 | F (2, 483) = 3.85,  *p =* .022 | 6.12  (2, 86.87) | .003 | 0ms –150ms (*p* = .02)  0ms –300ms (*p* = .005)  150ms –300ms (*p = .*70, ns) | |
| *Exp. 1B* | 0ms= 3.70  150ms= 2.90  300ms= 2.98 | W = 0.937,  p < .001 | F (2, 346) = 4.72,  *p =* .009 | 13.54  (2, 61.83) | <.001 | 0ms–150ms (*p* < .001)  0ms –300ms (*p* < .001)  150ms –300ms (*p = .84*, ns) | |
| *Exp. 2A* | 0ms= 3.63  150ms= 3.18  300ms= 2.97 | W = 0.930,  p < .001 | F (2, 343) = 8.17,  *p* < .001 | 5.80  (2, 63.88) | .005 | 0ms –300ms (*p* = .003)  0ms –150ms (*p* = .078, ns)  150ms –300ms (*p = .*46, ns) | |
| *Exp. 2B* | 0ms= 3.62  150ms= 3.10  300ms= 3.14 | W = 0.923,  p < .001 | F (2, 344) = 6.39,  *p =* .002 | 4.82  (2, 63.97) | .011 | 0ms –150ms (*p* = .024)  0ms –300ms (*p* = .025)  150ms –300ms (*p = .99*, ns) | |
| *Exp. 3* | 0ms= 3.65  150ms =3.18  300ms= 3.07 | W = 0.933,  p < .001 | F (2, 345) = 2.53,  *p* = .080 (ns) | 5.62  (2, 61.76) | .006 | 0ms –150ms (*p* = .025)  0ms –300ms (*p* = .006)  150ms –300ms (*p = .*80, ns) | |
